# Clinical and cost effectiveness of arthritis gloves in rheumatoid arthritis (A-GLOVES): randomised controlled trial with economic analysis

**DOI:** 10.1186/s12891-020-03917-8

**Published:** 2021-01-08

**Authors:** Alison Hammond, Yeliz Prior, Sarah Cotterill, Chris Sutton, Elizabeth Camacho, Calvin Heal, Jo Adams, Yvonne Hough, Terence W. O’Neill, Jill Firth

**Affiliations:** 1 Centre for Health Sciences Research, School of Health and Society, University of Salford, Allerton Building, Frederick Road, Salford, M6 6PU UK; 2 Centre for Biostatistics, Division of Population Health, Health Services Research and Primary Care, University of Manchester, Manchester, UK; 3 Manchester Centre for Health Economics, Division of Population Health, Health Services Research and Primary Care, University of Manchester, Manchester, UK; 4 Health Sciences, University of Southampton, Southampton, UK; 5 Rheumatology Occupational Therapy, St Helens and Knowsley Teaching Hospitals NHS Trust, St Helens, UK; 6 Centre for Epidemiology Versus Arthritis, Division of Musculoskeletal and Dermatological Sciences, Manchester Academic Health Science Centre, University of Manchester, Manchester, UK; 7 NIHR Manchester Biomedical Research Centre, Manchester University NHS Foundation Trust, Manchester, UK; 8 Pennine MSK Partnership, Oldham, Manchester, UK

**Keywords:** Rheumatoid arthritis, Rehabilitation, Hand, Pain, Orthotic devices, Clinical trial

## Abstract

**Background:**

Arthritis (or compression) gloves are widely prescribed to people with rheumatoid arthritis and other forms of hand arthritis. They are prescribed for daytime wear to reduce hand pain and improve hand function, and/or night-time wear to reduce pain, improve sleep and reduce morning stiffness. However, evidence for their effectiveness is limited. The aims of this study were to investigate the clinical and cost effectiveness of arthritis gloves compared to placebo gloves on hand pain, stiffness and function in people with rheumatoid arthritis and persistent hand pain.

**Methods:**

A parallel randomised controlled trial, in adults (≥ 18 years) with rheumatoid or undifferentiated inflammatory arthritis at 16 National Health Service sites in the UK. Patients with persistent hand pain affecting function and/or sleep were eligible. Randomisation (1:1) was stratified by recent change (or not) in medication, using permuted blocks of random sizes. Three-quarter-finger length arthritis gloves (Isotoner®: applying 23-32 mmHg pressure) (intervention) were compared to loose-fitting placebo gloves (Jobskin® classic: providing no/minimal pressure) (control). Both gloves (considered to have similar thermal qualities) were provided by occupational therapists. Patients and outcome assessors were blinded; clinicians were not. The primary outcome was dominant hand pain on activity (0–10) at 12 weeks, analysed using linear regression and intention to treat principles.

**Results:**

Two hundred six participants were randomly assigned (103 per arm) and 163 (84 intervention: 79 control) completed 12-week follow-up. Hand pain improved by 1.0 (intervention) and 1.2 (control), an adjusted mean difference of 0.10 (95% CI: − 0.47 to 0.67; *p* = 0.72). Adverse events were reported by 51% of intervention and 36% of control group participants; with 6 and 7% respectively, discontinuing glove wear. Provision of arthritis gloves cost £129, with no additional benefit.

**Conclusion:**

The trial provides evidence of no clinically important effect of arthritis gloves on any of the trial outcomes (hand pain, function and stiffness) and arthritis gloves are not cost-effective. The clinical and cost-effectiveness results support ceasing provision of arthritis gloves in routine clinical practice. Funding: National Institute for Health Research.

**Trial registration:**

ISRCTN, ISRCTN25892131; Registered 05/09/2016: retrospectively registered.

**Supplementary Information:**

The online version contains supplementary material available at 10.1186/s12891-020-03917-8.

## Introduction

Rheumatoid arthritis (RA) affects 1% of the world’s population [1]. Disease modifying anti-rheumatic drugs (DMARDs) are prescribed as soon as possible to control symptoms, including to those with persistent synovitis where other pathologies are ruled out but not yet meeting criteria for RA, i.e. undifferentiated inflammatory arthritis (UIA). Functional ability can still deteriorate even though disease activity is controlled [2]. Over 90% of people with RA and UIA report hand symptoms of pain, stiffness, muscle weakness, paraesthesia, and difficulty making a fist [3]. Most have bilateral hand symptoms, resulting in difficulties with work, everyday activities and leisure.

**Fig. 1:**
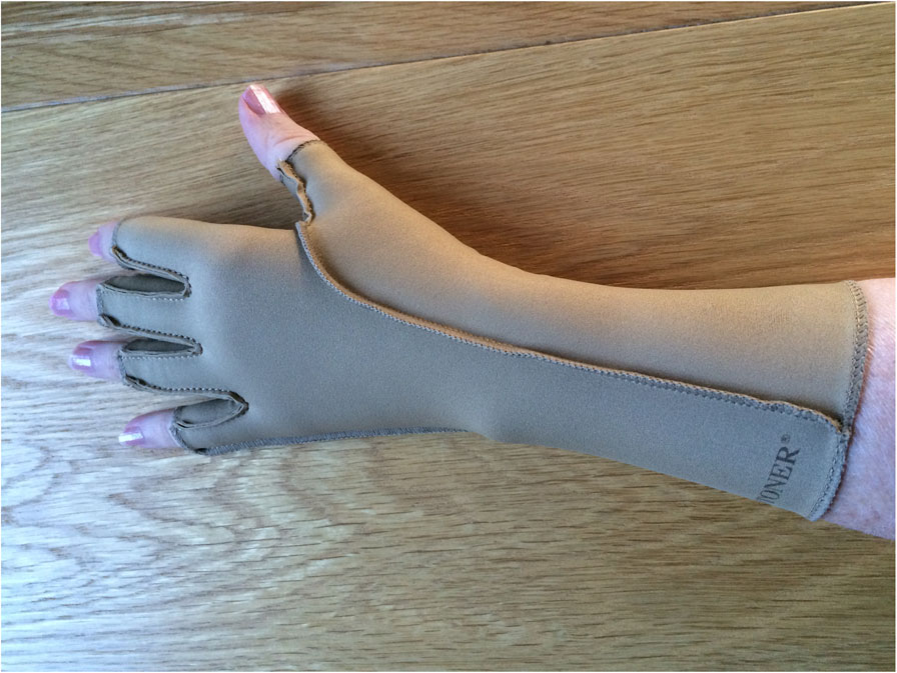
Intervention (Isotoner®) Arthritis Glove

Arthritis gloves (or compression gloves) have been commonly prescribed since the 1980’s for people with RA and UIA in the United Kingdom (UK), North America and Europe [4–7]. A survey of occupational therapists in the UK identified that the most common model and make prescribed are three-quarter length finger Isotoner® gloves (Fig. 1) [4]. They are prescribed for daytime wear to reduce hand pain and improve hand function, and/or night-time wear to reduce pain, improve sleep and reduce morning stiffness [4, 5]. The second most common are three-quarter length finger oedema gloves, of which there are several makes available [4]. The mechanism whereby arthritis gloves are thought to impact on hand symptoms is through compression, which: removes extracellular fluid, thus reducing pain, stiffness and improving finger movement; and increases blood flow, thus increasing warmth and reducing pain [6, 7]. In clinical practice, therapists ensure a “snug fit” around the proximal interphalangeal joints (PIPJs), metacarpophalangeal joints (MCPJs) and across the dorsum of the hand, i.e. to ensure enough compression is applied without restricting circulation to the fingers or causing pins and needles. It is unclear what a therapeutic level of compression is, as no physiological studies have been conducted. Manufacturers of commonly prescribed glove makes report pressures of 15 to 32 mmHg [8, 9]. The amount of compression applied depends on: the amount of elastane in the glove fabric; the individual’s hand size and shape; and the glove fit. Isotoner® gloves have the highest amount of elastane content of those currently on the market. According to the manufacturers’ specifications, Isotoner® gloves exert the highest pressure [8, 9].

Arthritis gloves are considered a comparatively low-cost, quickly provided treatment for pain relief, although they are a recurrent cost as they need replacing every six-months. Gloves are also commonly prescribed to people with other forms of inflammatory arthritis and with hand osteoarthritis. The global market for arthritis gloves is rising [10] and millions of people with hand arthritis worldwide purchase these gloves for themselves.

Systematic review evidence is inconclusive about the effectiveness of arthritis gloves [5]. Previous trials were small (*n* = 8 to 24), evaluated a variety of full-length finger arthritis gloves in RA for night-time use only [6, 7, 11–13], and had moderate to high risk of bias [5]. Three trials, with moderate risk of bias, showed significant reductions in PIPJ circumference. However, this was only 0.7 to 1.15 mm and it is questionable what the benefits of this were [5]. Trials were inconclusive about effects on nocturnal hand pain, morning stiffness and hand swelling. No benefits were identified in range of motion, dexterity, grip and pinch strength [5]. One trial found arthritis gloves and thermal gloves had similar effects in reducing nocturnal hand pain and hand stiffness [6]. Gloves’ effect on day-time hand pain, the most common reason for prescription nowadays, has not been evaluated.

We conducted a pre-test post-test feasibility trial to evaluate Isotoner® arthritis gloves, over a four-week period, in people with RA and UIA in 10 Rheumatology occupational therapy departments. In this we: developed a treatment manual, based on therapists’ expertise in providing arthritis gloves, to standardise delivery; developed an arthritis glove information sheet for participants; developed and tested trial procedures; identified the most relevant outcome measure considered by participants was day-time hand pain; and that this and other secondary outcomes deemed most relevant were self-report and therefore collectable using reliable, valid patient reported outcome measures. Participants reported they liked the warmth, comfort and gentle support provided by the arthritis gloves [14]. The results of this trial indicated that the procedures were feasible and there were some improvements in hand pain, stiffness and function, with the caveat that there was no control group. This supported the need for a definitive trial.

We sought the input of our clinical stakeholders, participating therapists and our patient and public involvement (PPI) representatives throughout this research. We conducted a series of focus groups to discuss the feasibility study results and design the A-Gloves trial, to ensure it would be acceptable to both patients and participating clinicians. We discussed what the comparator intervention should be: usual care; ordinary gloves plus usual care; or placebo gloves plus usual care. Our patient research partners emphasised the importance of comparing arthritis gloves with placebo gloves, and that these should be credible (i.e. another model of arthritis glove which was loosely fitted to apply no pressure but provide similar warmth) to ensure that the effects of wearing a “medical device” were controlled for. We discussed the length of follow-up required. The PPI group and therapists emphasised glove wear should increase gradually over the first few weeks, in order to become accustomed to glove effects. Therapists reported patients usually start to report any benefits, or adverse effects, by a four- week review appointment. As gloves are intended to be worn long-term, we selected a 12-week follow-up as: allowing several weeks for glove tolerance to develop; participants to experience effects of regular wear for up to 2 months across a range of activities; and being a feasible time-scale within the funding period. Therapists highlighted the importance of ensuring participating therapists understand that there is only low-level evidence about arthritis gloves currently, it is unclear if arthritis gloves are effective and therefore there is clinical equipoise between arthritis and placebo gloves (i.e. a placebo glove providing warmth is an ethically acceptable alternative). This was considered essential as the participating therapists all normally regularly provided arthritis gloves as part of usual care. Accordingly, we needed to facilitate therapists to overcome personal preferences to provide arthritis gloves, be ethically willing to enrol participants and provide placebo gloves in a trial, be able to provide placebo gloves in a credible way and not have prior expectations of the trial findings.

In the Arthritis Gloves (A-GLOVES) trial we aimed to investigate, for people with RA and UIA with persistent hand pain, the comparative clinical and cost-effectiveness of providing arthritis gloves or placebo gloves in addition to usual care.

## Methods

### Study design and ethics

The A-GLOVES trial was a pragmatic, multicentre, investigator-blind, parallel-group randomised controlled trial. The trial was conducted in rheumatology occupational therapy departments in 16 National Health Service sites in England and Scotland. The trial was approved by the North of Scotland National Research Ethics Service Committee (REC reference 15/NS/0077). The full protocol has been published [15].

### Participants

Patients were eligible for inclusion if aged 18 years or older, diagnosed with RA or UIA by a Rheumatology consultant, with persistent pain in the PIPJs or MCPJs causing at least one of: difficulty using the hands during the day (for day wear); disturbed sleep (for night wear); and limited ability to use the hands in the morning (for night wear). We excluded patients diagnosed with other inflammatory forms of arthritis affecting the hands (e.g. psoriatic arthritis, gout, ankylosing spondylitis); severe Raynaud’s disease, hand circulatory disturbances, hand neuropathies or hand deformities; and any contraindications to glove-wear (e.g. eczema, broken skin). Participants should not previously have worn arthritis gloves.

We asked clinicians to identify potentially eligible patients during clinic visits or from therapy records. Patients were given an invitation letter and information sheet. If interested they saw a research practitioner or occupational therapist to discuss the trial, check eligibility, complete consent and study registration and receive the baseline questionnaire (completed at home then mailed to the research co-ordinating centre). Participants at screening who had recently received a steroid injection or started oral steroids were deferred for 6 weeks after injection or drug start, and re-screened to check for eligibility. Steroids could be a confounding variable as they quickly improve hand symptoms [16].

### Randomisation and blinding

Participants were randomly assigned (1:1) to the intervention or control group, stratified by whether the participant had a change in or new medication (DMARDs or biologics) or not within the last 3 months, using permuted blocks of random sizes. Randomisation was completed by the Lancashire Clinical Trials Unit using Sealed Envelope, a web-based central randomisation service [17]. Participants were accumulated into both groups from start of recruitment at each site. After randomisation, allocation was unblinded to therapists delivering treatment. Participants were not blinded to group allocation. The study was described as comparing the effects of two types of arthritis glove without divulging the differences. Therapists were asked not to use the term “compression gloves” to participants, in order to reduce the risk of unblinding. Investigators and data management staff were blinded to group allocation. Data were analysed blinded to group allocation.

### Interventions

Following randomisation, a referral was sent to the treating therapist, including group allocation. Gloves were fitted within 3 weeks, with a review appointment 2 to 4 weeks later to check for glove fit and any adverse events. We tested the intervention gloves against placebo gloves, in order to control for therapist time, attention and effects of receiving a medical device.

Participants in the intervention group, received correctly fitted three-quarter length finger Isotoner® gloves. These are made of 80% nylon and 20% elastane. These were selected for testing as they exert the highest level of pressure of arthritis gloves available, at 23-32 mmHg [8]. If compression is the mechanism of action of arthritis gloves, then these gloves are more likely to lead to effects being detected. Additionally, as these are the most popular glove prescribed, testing Isotoner® gloves reflects UK clinical practice [4]. The size range comfortably fits hands up to 23.5 cm. MCPJs circumference. Clinically, fitting Isotoner® gloves is not possible for people with larger hands, as they are too tight and can cause problems with pins and needles, numbness or limit finger circulation (Fig. 1).

**Fig. 2:**
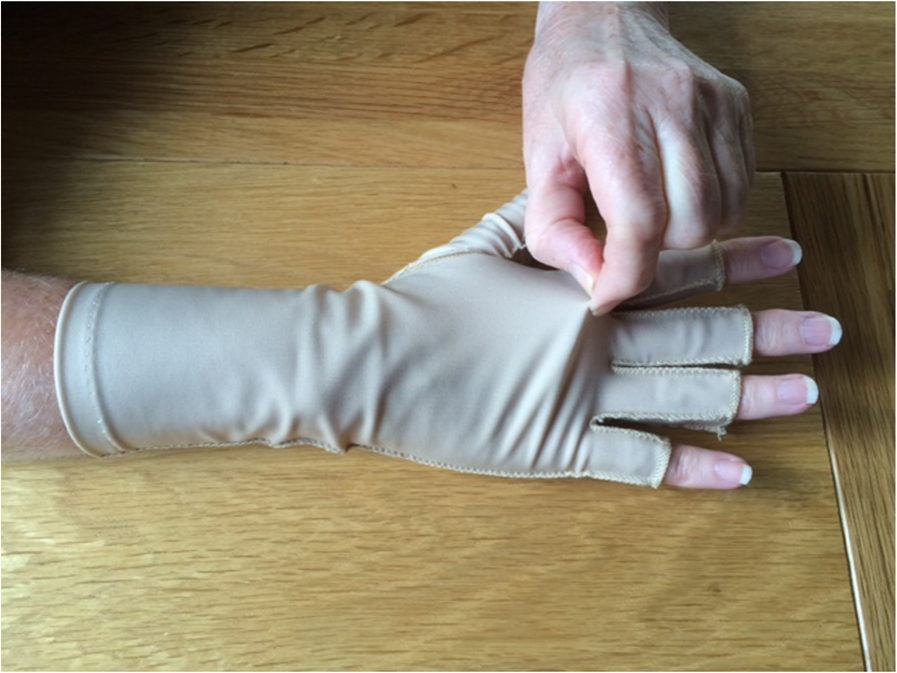
Placebo Glove (loose-fitting Jobskin® classic oedema glove: control group)

Participants in the control group received loose-fitting three-quarter length finger Jobskin® classic oedema gloves, made of 89% nylon and 11% elastane (placebo gloves). When fitted correctly, these exert 15-25 mmHg pressure [9]. However, these were fitted at least one size too large and exerted no pressure. A focus group of RA patients and expert rheumatology occupational therapists chose the Jobskin® gloves, worn loosely, as a credible placebo glove because of sufficiently similar appearance, material and warmth to the intervention gloves. Additionally, Jobskin® gloves have the largest size range available of arthritis glove makes prescribed in clinical practice, accommodating hands up to 25.4 cm. MCPJs circumference (Fig. 2).

At the first appointment, therapists measured the participant’s MCPJs circumference to determine correct glove size, visually checked appropriate fit and discussed hand symptoms and function to determine an individual wear regimen. Participants in either group, with hands larger than 23.5 cm MCPJs circumference, were not fitted with gloves as: the Isotoner® intervention gloves would be too tight; and the largest size of placebo glove insufficiently loose. Previous studies have reported difficulties fitting gloves for those with large hands, with some men reporting gloves being too short or small [5]. Most participants were issued with gloves for both hands for day and night wear. However, some were provided with only one glove (usually the dominant hand); or advised to wear gloves during the day or night only, i.e. consistent with routine clinical practice. Participants could wear gloves for most of the day and all night but informed not to wear 24 h a day. Participants were given booklets about hand self-management, including joint protection [18], and hand exercise [19] and asked to do hand exercises daily. We have made available the Arthritis Gloves Occupational Therapist Provision Manual describing the clinical protocol [20].

Participants continued to receive their Rheumatology and Occupational Therapy department’s usual care. The therapist could provide advice and training for up to 1 h in joint protection and hand exercises when providing gloves, (if needed, if the participant had not already received these), reflecting clinical practice [4, 14]. Other therapies could be provided.

#### Therapist training

Prior to the trial starting, we explained to therapists: the existing research about arthritis gloves, the lack of good quality and contradictory evidence for efficacy and effectiveness (clinical equipoise); the trial design to ensure that they understood the reasons for testing arthritis against placebo gloves and that warmth might be a contributory mechanism, meaning placebo gloves are an acceptable comparator; and discussed any concerns about providing placebo gloves. Therapists agreed to take part following consideration of the trial design. Prior to attending the trial training course, therapists were asked to read two articles: a systematic review of arthritis gloves [5] and an article exploring therapists’ views about placebo splint provision within a trial [21]. They then attended a one-day training course including: education about randomised controlled trials; the A-Gloves trial procedures, theoretical and practical training in intervention and placebo glove fitting, in order to standardise the interventions; and a focus group about their views of providing placebo gloves and personal, service and research methodology issues in relation to their trial participation [22]. Training explained the placebo effect and emphasised: the importance of ensuring placebo gloves were a loose fit so that no pressure was being applied, to avoid treatment contamination in the control group; and how to answer control participants’ queries related to the loose-fit of the placebo gloves, in order to reduce the risk of control participant stopping glove-wear. The accompanying glove provision manual detailed procedures, glove-fitting and issues of providing placebo gloves [20].

### Data collection

Baseline data were collected using a postal self-completed questionnaire. Randomisation occurred immediately after receipt. Follow-up data were obtained 12-weeks post-randomisation by postal self-completed questionnaire. After 1 week, participants were reminded by e-mail, text or telephone to return the questionnaire. If no response was received: after 2 weeks, participants were sent a second copy of the questionnaire; and after 4 weeks they were contacted by telephone to obtain a minimal data set, if possible.

### Outcomes

The primary outcome measure, at 12-weeks post-randomisation, was hand pain in the dominant hand during the daytime when doing moderate hand activities (e.g. housework, cooking, DIY, gardening), measured on a numeric rating scale (NRS), with anchor points of no pain (0) and severe pain (10). Minimal clinically important differences (MCID) for pain scales in RA are 1.1 points on a 0–10 NRS [23, 24]. Hand pain was selected because it is the most common reason therapists provide gloves and the symptom that patients most frequently describe as benefiting from glove use [5, 6, 14].

Secondary outcomes were: non-dominant hand pain in the day (0–10 NRS), dominant and non-dominant hand pain at night (0–10 NRS); hand stiffness (0–10 NRS), owing to a lack of predefined MCID this was considered as ≥ 0.5 SD of mean baseline score [25, 26], i.e. in this trial = 1.4 points); duration of hand early morning stiffness (minutes); hand function (Measure of Activity Performance-Hand, with higher scores denoting worse hand function and Minimal Detectable Change (at 95% confidence interval: MDC_95_) of 3.99 [27]; and Michigan Hand Outcomes Questionnaire, with higher scores denoting better hand performance (apart from the hand pain sub-scale), with an MCID of 13 points [28, 29]; and disability (Health Assessment Questionnaire, with higher scores denoting worse function [30]. Participants completed the EQ-5D-3L [31] as a measure of health benefit for the economic evaluation and reported their use of health and social care services at baseline and follow-up. Additionally, at 12-week follow-up, we asked participants about: glove use; perceptions of glove-wear; and new steroid prescription (injection or oral). Therapists recorded glove provision, wear regimen and occupational therapy provided as part of usual care on standardised treatment logs. These were reviewed to identify documentary evidence of glove provision, treatment duration, and any adverse events. A detailed description of outcome measures was published in the trial protocol [15]. Those questionnaire items developed specifically for the trial were constructed with the assistance of the patient research partners (see study questionnaire in Supplementary Materials).

### Sample size

Using data from a pilot study [14], the mean change in hand pain NRS (measured 4 weeks post-intervention) was − 1.03 (SD 2.22). The 80% upper one-sided confidence limit of the estimated SD, i.e. 2.48 was used. To identify a MCID of 1.1-point, SD = 2.48, significance level of 0.05 and 80% power, 80 participants per group were required. Allowing for 22% missing 12-week primary outcome data, the target sample size was 205 randomised participants.

### Statistical and economic analysis

The analysis followed a pre-specified statistical analysis plan and was by intention to treat, without imputation of missing data. STATA V.14 was used [32]. Baseline characteristics were described, overall and by group, reporting mean (standard deviations), median (IQR) or number (proportion), as appropriate. Primary effectiveness analysis used linear regression to estimate an adjusted mean difference comparing dominant hand pain during activity at 12 weeks (primary outcome) between groups, adjusting for baseline scores and the stratification variable (recent DMARD changes or not). Secondary analyses used appropriate modelling approaches (multiple linear regression, logistic regression or ordinal logistic regression), to estimate the effect of group allocation on the other health outcomes at 12 weeks. A sensitivity (per protocol) analysis omitted those who did not receive gloves, or who self- reported being prescribed steroids (oral or injection) during the trial. Data were analysed by person and using dominant hand results, as reported by the participant.

The economic analysis compared the costs and health benefits of the gloves from a health and social care perspective. The primary economic analysis was based on 151 complete cases (i.e. participants with no missing data). Sensitivity analysis explored the cost effectiveness of arthritis gloves using the primary outcome measure, in the sub-group of participants who were treated as per the trial protocol (i.e. received gloves), and under alternative models of glove provision.

Intervention costs were estimated from individual patient treatment logs, for the number of gloves provided, assuming one visit to an occupational therapist. Published unit costs were used to calculate total costs for the intervention and healthcare utilisation in both groups [33, 34]. Quality-adjusted life-years (QALYs) were estimated from the EQ-5D-3L data, using an area under the curve approach and published utility tariffs for England [35]. Linear regression analysis was used to estimate net QALYs and a generalised linear model with log link and gamma family was used to estimate net costs for the arthritis gloves compared to the placebo gloves, adjusted for baseline values and stratification variable. Bootstrapping (*n* = 10,000 simulations) was used to estimate the probability that arthritis gloves were cost-effective at different willingness to pay thresholds (WTPTs).

## Results

### Recruitment

**Fig. 3:**
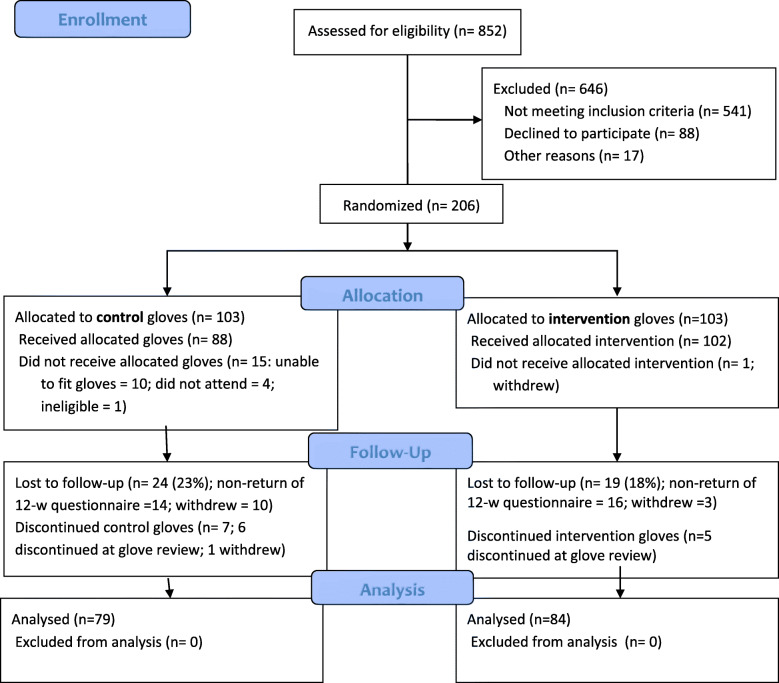
Workwell CONSORT Flow Diagram

Figure 3 shows the patient flow through the trial. Between February 2016 and May 2017, 206 participants were recruited and randomised, with 103 in the intervention and 103 in the control groups. Within the intervention group, 102 (99%) received Isotoner® gloves. Within the control group, 88 (85%) received the placebo gloves: 10 could not be fitted with gloves due to larger hand size. At 12-week follow-up, data were received from 84 (82%) in the intervention and 79 (77%) in the control group. Those not fitted with gloves continued to be followed up (Supplementary Table S1).

### Participants

The median age of participants was 59 years, over 80% were women and a third employed. Most were diagnosed with RA (86% control; 84% intervention). Median time since diagnosis was 4 years and most (90%) were prescribed DMARDs or biologic drugs (Table 1).

**Table 1:** Baseline participant characteristics of the intention-to-treat population (*n* = 206)

	Control (***n*** = 103)	Intervention (***n*** = 103)
Age (years): median (IQR)	60 (51,68)	58 (51,67)
Sex: female (%)	82 (80%)	84 (82%)
Diagnosis:		
RA	89 (86%)	87 (84%)
UIA	14 (14%)	16 (16%)
Hand dominance:		
Right (%)	92 (89%)	93 (90%)
Left (%)	9 (9%)	3 (3%)
Both (%)	2 (2%)	7 (7%)
Living status: Alone (%)	18 (17%)	19 (18%)
Living with children: yes (%)	10 (10%)	14 (14%)
Employment status:		
Retired	50 (49%)	44 (43%)
Due to ill health	9 (9%)	18 (17%)
Other	41 (40%)	26 (25%)
Full-time work	24 (24%)	21 (20%)
Part-time work	13 (13%)	18 (17%)
Homemaker	9 (9%)	6 (6%)
Long-term sick	2 (2%)	7 (7%)
Unemployed	3 (3%)	3 (3%)
Self-employed	1 (1%)	3 (3%)
Student	0 (0%)	1 (1%)
Missing	1. (1%)	
Symptom duration (months):		
Median (IQR)	60 (24,168)	72 (18,174)
Diagnosis duration (months):		
Median (IQR)	48 (10,144)	51 (8,168)
Medication regimen:		
0 DMARDs ^a^	10	11
1 DMARD	51	56
2 or more DMARDs	42	36
Biologics ^b^	15	18
Started new/ changed DMARD or biologic in previous 3 months ^c^: yes (%)	34 (33%)	34 (33%)

^a^*DMARD* Disease Modifying Anti-Rheumatic Drug; ^b^participants were also prescribed a DMARD; ^c^stratification variable

### Glove provision and other therapy

Most participants (78%) received gloves within 3 weeks of referral. All received gloves for their dominant hand, and most in both groups for both hands (95/102 (93%) intervention; *n* = 83/88 (94%) control). In both groups, 73% were recommended to wear gloves during the day. Two-thirds of those responding at 12-weeks answered the adherence items. For the remainder, glove wear was unknown.

Self-reported glove wear was very similar between groups for both hands at on average: 5 h during the daytime on 5 days/ week; and 6 h at night for 5 nights/week (Table 2). On average, the intervention group wore arthritis gloves somewhat less often at night, compared to placebo glove wear in the control group. At 12-week follow-up, there were no differences between groups in treatment duration or receiving steroids (Supplementary Table S2).

**Table 2:** Recommended glove-wear regimen, self-reported glove wear and glove benefit (12- week follow-up: *n* = 154)

	Control (***n*** = 70^**a**^):	Intervention (***n*** = 84):	***p***
Recommended glove-wear regimen:			
- Day time only	9 (13%)	20 (24%)	0.08
- Day and night	42 (60%)	41 (49%)	0.17
- Night-time only	19 (27%)	23 (27%)	0.97
Number of days gloves worn (day-time) per week in last 4 weeks: mean (SD)			
- Dominant hand	5.2 (1.8) (*n* = 47)	5.3 (1.8) (*n* = 56)	0.80
- Non-dominant hand	5.2 (1.9) (*n* = 45)	5.2 (1.8) (*n* = 53)	0.94
Number of minutes gloves (worn (day-time) per day in last 4 weeks: Mean (SD)			
- Dominant hand	310.0 (218.9) (*n* = 48)	316.9 (215.1) (*n* = 57)	0.87
- Non-dominant hand	317.6 (225.5) (*n* = 46)	325.1 (226.5) (*n* = 54)	0.87
Number of nights gloves worn per week in last 4 weeks: mean (SD)			
- Dominant hand	5.5 (1.9) (*n* = 52)	4.9 (2.1) (*n* = 55)	0.14
- Non-dominant hand	6.0 (1.5) (*n* = 48)	5.1 (2.1) (*n* = 56)	**0.01**
Number of minutes gloves worn per night in last 4 weeks: Mean (SD)			
- Dominant hand	412.6 (131.7) *n* = 53	384.4 (136.5) *n* = 58	0.27
- Non-dominant hand	419.4 (140.0) *n* = 50	395.8 (140.1) *n* = 58	0.38
Self-reported glove perceptions:			
- Gloves are beneficial	51 (73%)	61 (73%)	0.97
- Will continue to wear gloves	50 (72%)	59 (72%)	0.94
	(*n* = 51)	(*n* = 61)	
- Gloves give warmth	41 (80%)	45 (74%)	0.41
- Gloves give comfort	38 (75%)	52 (85%)	0.15
- Sleep better	21 (41%)	25 (41%)	0.98

^a^10 control participants could not be fitted with gloves

### Primary outcome

At baseline both groups had moderate day-time levels of dominant hand pain during activity (6.3 on the 0–10 NRS). At 12-week follow-up (*n* = 154), day-time dominant hand pain reduced in both groups, with the intervention group (*n* = 84) reducing slightly less than the control group (*n* = 79) (1.0 versus 1.2 points). There was no evidence of a difference between the intervention and control gloves, after adjusting for the stratification variable and baseline pain scores. The adjusted difference of 0.1 (favouring the control group) between groups (95% CI − 0.47 to 0.67) was not statistically significant nor clinically relevant (as the largest plausible positive effect of the intervention gloves was 0.47 units, less than half the MCID of 1.1 points) (Table 3). In the sensitivity (per protocol) analysis (i.e. only those receiving the correct gloves and who did not receive steroids), there was also no statistically significant difference between groups (0.2 points, CI − 0.41 to 0.81; *p* = 0.51). No further analysis adjusting for effects of glove-wear was conducted. As there were no substantive differences in self-reported frequency of dominant hand glove wear between groups, this would have minimal impact on the estimated between group differences in the primary outcome.

**Table 3:** Comparative effectiveness of intervention and placebo gloves at 12 weeks

	Control			Intervention				
	Baseline Mean (S.D.) Control *n* = 103	12-week follow-up. Mean (S.D.) Control *n* = 79	Score Change (0-12w)	Baseline Mean (S.D.) *n* = 103	12-week follow-up. Mean (S.D.) *n* = 84	Score Change (0-12w)	Adjusted between group difference ^e^ (95% CI)	*p*-value
Dominant hand pain during activity (0–10 NRS ^a^)	6.3 (2.2)	5.1 (2.3)	1.2	6.3 (1.9)	5.3 (2.3)	1.0	0.1 (−0.47, 0.67)	0.72
Non-dominant hand pain during activity (0–10 NRS)	5.6 (2.4)	4.5 (2.4)	1.1	5.7 (2.4)	4.3 (2.5)	1.4	−0.29 (0.94, 0.36)	0.38
Dominant hand pain during rest (0–10 NRS)	5.2 (2.4)	3.8 (2.4)	1.4	4.6 (2.2)	3.9 (2.2)	0.7	0.29 (−0.32, 0.90)	0.35
Non-dominant hand pain during rest (0–10 NRS)	4.7 (2.7)	3.6 (2.4)	1.1	4.4 (2.5)	3.3 (2.3)	1.1	−0.15 (−0.81, 0.51)	0.66
Dominant hand pain at night (0–10 NRS)	5.5 (2.8)	3.8 (2.6)	1.4	5.0 (2.5)	4.0 (2.5)	1.4	0.34 (−0.30,0.98)	0.30
Non-dominant hand pain at night (0–10 NRS)	4.9 (2.9)	3.5 (2.7)	1.4	4.6 (2.8)	3.2 (2.5)	1.4	−0.32 (−0.98,0.35)	0.35
Hand stiffness in the morning (Minutes)	141.1 (272.8)	96.4 (226.7)	44.7	132.7 (269.8)	62.5 (53.0)	70.2	32 (−0.25,15.83)	0.37
Hand stiffness dominant hand (0–10 NRS)	6.0 (2.6)	4.9 (2.6)	1.1	5.6 (2.9)	4.9 (2.9)	0.7	0.28 (−0.41,0.97)	0.42
Hand stiffness non-dominant hand (0–10 NRS)	5.4 (2.9)	4.1 (2.7)	1.3	5.2 (2.9)	4.2 (2.9)	1.2	0.17 (−0.55,0.89)	0.64
Self-reported dominant hand condition (1–5)	3.3 (0.7)	2.9 (0.8)	0.4	3.3 (0.7)	2.8 (1.0)	0.5	−0.18 (−0.77, 0.41)	0.56
Overall MAPHAND ^b^ (Hand function) (0–3)	1.4 (0.6)	1.2 (0.6)	0.2	1.5 (0.5)	1.2 (0.6)	0.3	−0.05 (− 0.18, 0.07)	0.39
Overall HAQ ^c^ (0–3)	1.5 (0.7)	1.3 (0.7)	0.2	1.5 (0.6)	1.4 (0.8)	0.1	0 (−0.13,0.13)	0.99
Overall MHQ ^d^ (0–100)	49.8 (13.7)	57.2 (17.1)	7.4	49.3 (11.2)	57.0 (17.0)	7.9	0.37 (−3.45, 4.20)	0.85
**-** Overall hand function	42.8 (18.2)	50.3 (22.8)	7.5	43.6 (14.8)	54.6 (19.9)	11	3.49 (−2.05, 9.04)	0.22
**-** Activities of daily living	51.1. (25.8)	59.7 (27.4)	8.6	51.2 (24.0)	59.0 (27.1)	7.8	−0.69 (−6.10; 4.71)	0.80
**-** Work	39.5 (23.7)	50.9 (23.1)	11.4	37.7 (19.9)	48.5 (26.0)	10.8	−1.30 (−7.28, 4.69)	0.67
**-** Pain	35.1 (25.8)	33.6 (23.4)	1.5	32.7 (24.6)	33.1 (23.4)	0.4	−0.07 (− 6.54, 6.40)	0.98
**-** Aesthetics	65.5 (19.5)	69.3 (21.4)	3.8	64.2 (18.5)	66.4 (22.7)	2.2	−2.18 (−8.08, 3.72)	0.47
**-** Satisfaction	35.3 (21.5)	48.5 (26.6)	13.2	32.3 (16.3)	48.4 (25.4)	16.4	1.95 (−4.99, 8.90)	0.58

^a^*NRS* Numeric Rating Scale, ^b^*MAPHAND* Measure of Activity Performance – Hand, ^c^*HAQ* Health Assessment Questionnaire, ^d^*MHQ* Michigan Hand Questionnaire, ^e^adjusted between group difference – adjusted for stratification variable (change in DMARDS) and baseline score

### Secondary outcomes

Both groups reported similar small levels of improvement in hand symptoms of non-dominant hand pain on activity, dominant and non-dominant hand pain at night, hand stiffness, duration of morning hand stiffness, and self-reported hand status, with no statistically significant or clinically important differences between groups. Both groups reported similar small improvements in hand function and disability and with no statistically significant or clinically relevant differences between groups (Table 3).

In both groups, in those reporting perceived effects, over 70% considered wearing gloves gave warmth and comfort, were beneficial, and they would continue to wear them (Table 2), supporting the qualitative study [36]. Forty-one per cent in both groups thought they helped them sleep better. Perceptions of glove-wear will be reported in detail elsewhere.

### Adverse events

In the intervention group, 47 (51%) reported an adverse event compared to 29 (36%) in the control group, with the most common in both groups being sleep disturbance as the gloves made hands feel hot and itchy. The intervention group reported more adverse events of pins and needles, numbness or fingertip discolouration (26 events) than the control group (7 events) (Supplementary Table S3). Similar numbers discontinued glove-wear at the glove review appointment due to adverse events (*n* = 7 (7%) control; *n* = 6 (6%) intervention, Fig. 3).

### Cost-effectiveness

Both control and intervention groups had the same health utility at baseline and accrued the same number of QALYs during follow-up (Table 4).

**Table 4:** Mean (SD) health utility, Quality Adjusted Life Years (QALYs) and costs

	Control			Intervention		
EQ-5D-3L	Baseline ***n*** = 103	12-week follow-up ***n*** = 79	Change ***n*** = 79	Baseline ***n*** = 103	12-week follow-up ***n*** = 84	Change ***n*** = 84
Utility (Mean, SD)	0.44 (0.37)	0.55 (0.55)	0.09 (0.27)	0.44 (0.34)	0.54 (0.31)	0.05 (0.26)
QALYs (Mean, SD)	0.12 (0.07)			0.12 (0.07)		
**Costs during follow-up (Mean, SD)**						
Outpatient	£255 (268) (*n* = 78)			£317 (333) (*n* = 82)		
Primary and community care	£59 (86) (*n* = 75)			£66 (101) (*n* = 81)		
Intervention	£0			£129 (*n* = 78)		
TOTAL^a^	£391 (543) (*n* = 75)			£552 (464) (*n* = 78)		

^a^ Total cost includes inpatient admissions, day case visits, and A&E visits. The mean intervention cost is reported for participants with complete cost data. Intervention cost comprised of actual number of compression gloves provided per participant costing £31.43/glove (NHS dispensing costs) and one visit to a secondary care occupational therapist costing £65.85 (NHS reference costs)

Use of physiotherapy services was higher in the intervention group (Supplementary Table 4). Costs associated with healthcare services used are shown in Table 4. The cost of providing placebo gloves is not included as these would not normally be provided in the NHS. The main difference in costs between groups was that of providing the intervention gloves. The results of the incremental cost-effectiveness analysis are reported in Table 5. The intervention gloves are associated with higher costs but comparable benefits to the placebo gloves and therefore unlikely to be cost-effective. The gloves had an incremental cost-effectiveness ratio (ICER) of £83,700 per QALY gained. The intervention gloves have a probability of 0.19–0.29 of being cost-effective if decision-makers are willing to pay £20–30,000/QALY, respectively. Sensitivity analyses confirmed that the intervention gloves are not likely to be cost-effective (results reported in full in Supplementary Table S5).

**Table 5:** **Results of incremental cost-effectiveness analysis for intervention versus control gloves**

	Net cost (95% CI)	Net QALY (95% CI)	ICER (£/QALY)	Probability intervention is cost effective versus control if WTPT =		
				£20,000/ QALY	£30,000/ QALY	£60,000/ QALY
Complete cases (ITT) (*n* = 151)	£251 (106, 396)	0.003 (−0.017, 0.023)	£83,700/QALY	0.19	0.29	0.44

Note: whole £ reported in table but ICERs calculated including pence

Covariates costs: pre-baseline costs, stratification variable

Covariates QALYs: baseline health status, stratification variable

*ICER* incremental cost-effectiveness ratio, *WTPT* willingness to pay threshold, *ITT* intention to treat

^a^at different willingness to pay thresholds, based on 10,000 bootstrap simulations

## Discussion

These results showed that, for people with RA or UIA, with moderate to severe hand pain, correctly fitted arthritis gloves led to slight improvements in hand pain (day or night), stiffness and function. However, wearing loose-fitting placebo gloves led to similar slight benefits. Improvements from both types of gloves were at, or only marginally above, the MCIDs for hand pain and hand function, and below for hand stiffness, with no significant or clinical differences between glove types. A high number experienced adverse events: a half of those wearing intervention and a third wearing control gloves. Both gloves led to similar levels of reporting about disrupted sleep. The intervention group experienced more neurological and circulatory adverse events resulting from the higher pressure applied by Isotoner® gloves. Self-reported glove wear was similar between groups, apart from the Isotoner® gloves being worn somewhat less at night by the intervention group. These neurological and circulatory adverse events are more likely to occur during prolonged periods of wear, such as at night. There was also some more reporting of hands becoming hot and itchy at night (especially in hot weather) in the intervention group. Both effects may have contributed to Isotoner® gloves being worn less often at night in the intervention group. The arthritis gloves were not cost-effective. Our results indicate arthritis gloves should not be provided in routine clinical practice. This would lead to a considerable cost saving per year to Rheumatology and Therapy departments, especially as glove provision is a recurrent cost.

Whilst we did not test arthritis gloves in people with other forms of hand arthritis, it would be reasonable to assume similar results. Arthritis gloves are recommended by arthritis charities and medical information websites [37, 38] and commonly purchased by people with hand arthritis. Such recommendations should be reviewed. Health professionals should enable people with hand arthritis to consider carefully, informed by the evidence, whether to purchase gloves for themselves.

Even though the results demonstrated little benefit in hand symptoms and hand function from wearing either gloves, most people in both groups thought they were beneficial and would continue to wear them. Both groups thought gloves gave warmth, comfort and support [36]. Arthritis gloves are thought to impact on hand symptoms through applying pressure. The results suggest that pressure is not an active ingredient in arthritis gloves, as loose-fitting gloves led to similar results. Perceived benefits were more likely due to warmth. A previous trial concluded arthritis gloves and thermal gloves had similar effects [6]. We hypothesize that the tactile feedback from wearing gloves provided the reported feelings of comfort and support and may sub-consciously have reminded users to take more care of their hand joints and subtly alter hand use during wear. A similar effect was also suggested in a pilot trial of thumb splints versus placebo thumb splints, which participants liked equally [39].

As both gloves gave similar results, and participants considered a main benefit was warmth [36], this suggests ordinary light-weight three-quarter finger gloves, made of nylon, cotton or wool (typically containing 5% elastane), purchased from High Street or online stores, could have similar effects. Therapists could recommend patients purchase such gloves instead. The time saved on glove provision could be used teaching hand exercises and joint protection, both of which are effective in reducing hand symptoms [40, 41]. Future research could investigate whether people with arthritis consider wearing ordinary light-weight gloves affects hand status.

This is the first randomised controlled trial to evaluate arthritis glove-wear on daytime hand pain and function, which are the main reasons for arthritis gloves being prescribed currently in routine clinical practice. The results also confirmed previous smaller trials of full-finger arthritis gloves that indicated there are no differences in nocturnal pain and stiffness between wearing arthritis or placebo gloves [5]. This trial demonstrates the importance of testing widely used interventions, long established in clinical practice, for which there is little evidence. Identifying that arthritis gloves are not effective is a positive finding, as it enables clinical practice to be evidence-based. Guidelines for the provision of hand orthoses in arthritis should be updated. This trial also provides sound evidence on which patients can base their own decisions as to whether to purchase arthritis gloves or ordinary gloves instead, as the latter would be a considerable cost-saving, especially for those with limited incomes.

The strengths of our trial include the large sample size, longer follow-up than previous trials (12 weeks compared to 4 weeks) and full economic evaluation. Recruiting from 16 rheumatology out-patient departments increased the likelihood of a representative sample. The trial design was developed with the PPI group’s and clinical therapists’ inputs to ensure the control intervention was credible and procedures feasible. We ensured before the trial and during training that therapists understood about current evidence and the clinical equipoise between arthritis and placebo gloves, discussed any ethical concerns, and they were willing and able to credibly provide the placebo gloves. The self-reported glove adherence rates in both groups were similar indicating training supported therapists in placebo glove provision. We tested the make of arthritis glove known to provide the highest level of compression in order to maximise the ability to detect any positive effects on outcomes. It is therefore unlikely that any other make of arthritis glove providing less compression would result in different outcomes. The limitations of this trial are, like many non-pharmacological trials, that therapists could not be blinded. We did not have a third usual care only control group. We considered it important to offer treatment to all participants meeting trial criteria. An objective independent clinical hand assessment (e.g. of hand swelling and hand function) was not included. However, our feasibility study indicated the main changes were in pain, stiffness and daily hand function, which are reliably measurable through patient reported outcomes [14].

## Conclusion

Arthritis gloves providing pressure and warmth and loose-fitting placebo gloves providing warmth, had only minimal effects on hand pain and function, with no differences between glove types. Participants perceived warmth as a main benefit from both gloves. Arthritis gloves were not cost-effective. Given these results, therapists could recommend patients buy ordinary three-quarter finger length gloves. These are widely available, would save health services money and be low-cost for patients. These findings inform evidence-based treatment choices for clinicians in specialist musculoskeletal services, and community services, and for patients considering purchasing arthritis gloves.

## Supplementary Information

**Additional file 1: Tables S1 to S5**. A-Gloves 12-week follow-up questionnaire

## Data Availability

The datasets used and/or analysed during the current study are available from the corresponding author on reasonable request. Clinical and trial procedures are described in the A-GLOVES: Occupational Therapy Glove Provision manual v2, freely available at http://usir.salford.ac.uk/42272

## Abbreviations

A&E: Accident and Emergency
A-GLOVES: Arthritis Gloves trial
CI: Confidence interval
DMARDs: Disease modifying anti-rheumatic drugs
HAQ: Health Assessment Questionnaire
ICER: Incremental cost-effectiveness ratio
IQR: Inter-quartile range
ITT: Intention to treat
MCPJs: Metacarpophalangeal joints
MAPHAND: Measure of Activity Performance Hand
MHQ: Michigan Hand Outcomes Questionnaire
MCID: Minimal clinically important difference
MDC: Minimal Detectable Change
NHS: National Health Service
NRS: Numeric rating scale
PPI: Patient and public involvement
PIPJs: Proximal interphalangeal joints
QALY: Quality Adjusted Life Year
RA: Rheumatoid arthritis
SD: Standard deviation
UIA: Undifferentiated inflammatory arthritis
UK: United Kingdom
WTPT: Willingness to pay threshold
