# Supplementary material for: Clinical and cost effectiveness of arthritis gloves in rheumatoid arthritis (A-GLOVES): randomised controlled trial with economic analysis
Source: BMC Musculoskelet Disord. 2021 Jan 8;22:47. doi: 10.1186/s12891-020-03917-8 (PMC7792116; doi:10.1186/s12891-020-03917-8)
Supplement: Supplementary file 1 — Additional file 1: Tables S1 to S5. A-Gloves 12-week follow-up questionnaire [file 12891_2020_3917_MOESM1_ESM.docx]

**Supplementary Materials:**

Supplement to Hammond A, Prior Y, Cotterill S, Sutton C, Heal C, Camacho E, Jones W, Adams J, Hough Y, O’Neill T, Firth J. Clinical and cost effectiveness of arthritis gloves in rheumatoid arthritis (A-GLOVES): randomised controlled trial with economic analysis. BMC Musculoskeletal Disorders 2020

**Supplementary Table S1: At screening, reasons why patients were ineligible; or unwilling, if eligible**

| **Ineligible:** | **n= 541^a^** | **Reasons why unwilling to participate, if eligible** | **n =88** |
| --- | --- | --- | --- |
| Previously worn arthritis gloves | 287 | Did not want to attend Occupational Therapy as interfering with work/daily routines | 9 |
| No persistent pain in PIP/MCPs joints | 97 | Unwilling to take part in research | 9 |
| Unable to read and understand English | 58 | Unwilling to complete questionnaire | 5 |
| Unwilling to wear arthritis gloves for 12 weeks | 47 | No reason given | 22 |
| Not diagnosed with RA or IA | 46 | Other reasons | 43 |
| Diagnosed with other rheumatic conditions | 36 |  |  |
| Severe hand deformities meaning unable to wear gloves | 26 | **Not progressed after consent** | **n=17** |
| Severe neuropathy in hands | 14 | Non-return baseline questionnaire | 1 |
| Severe Raynaud’s / other circulatory problem | 7 | Withdrawn prior to randomisation | 16 |
| Reason not recorded | 2 |  |  |

^a^ more than one reason for ineligibility could be recorded

**Supplementary Table S2: Treatment duration and other therapy provided**

|  | Control | Intervention | p-value |
| --- | --- | --- | --- |
| Treatment duration (minutes: mean (SD)):   - Initial glove appointment | 35.66 (17.93)  (n=99 ^a^) | 33.33 (12.60)  (n=103) | 0.29 |
| - Glove review appointment | 18.47 (10.09)  (n=78) | 16.88 (9.72)  (n=90) | 0.30 |
| Other interventions ^b^:  (minutes: mean (SD) | 24.35 (18.17)  (n=23) | 18.21 (18.11)  (n=29) | 0.23 |
| - Hand exercises | 10 | 7 |  |
| - Joint protection | 6 | 6 |  |
| - Assistive device recommendations | 5 | 8 |  |
| - Splint provision or repair | 3 | 7 |  |
| - Activities of daily living training | 0 | 2 |  |
| - Psychological intervention | 3 | 1 |  |
| - Medication/flare management advice | 2 | 1 |  |
| - Sleep/fatigue management | 1 | 1 |  |
| - Work advice | 1 | 0 |  |
| - Housing advice | 1 | 0 |  |
| Total treatment time (minutes: mean (SD) | 55.52 (27.75)  (n=99) | 53.01 (24.61)  (n=103) | 0.50 |
| New steroid use:   - Oral steroids started - Intramuscular or intra-articular injections | 2  12 | 2  10 | 0.54 |
| ^a^ Four control participants did not attend;  ^b^ Participants could receive more than one intervention. | | | |

**Supplementary Table S3: Frequency of adverse events reported from glove wear (n=76 reporting adverse events)**

|  | Control  (n=29) | Intervention  (n=47) |
| --- | --- | --- |
| Sleep disturbance at night (e.g. because the gloves made hands hot or itchy). | 13 | 17 |
| Pins and needles | 4 | 12 |
| Numbness | 2 | 8 |
| Fingertips became discoloured | 1 | 6 |
| Skin irritation | 3 | 4 |
| Other: | 10 | 10 |
| - Uncomfortable to wear due to hot weather | 6 | 6 |
| - Hand symptoms worsened | 3 | 3 |
| - Uncomfortable: reason not stated | 1 | 1 |
| Total | 43 | 67 |

**Supplementary Table S4: Use of healthcare resources during 12-week follow-up and mean usage for key services ^a^**

|  | **Control**  **(n=78)** | **Intervention**  **(n=84)** |
| --- | --- | --- |
|  | Participants reporting any use of any service, by setting  n/N (%) | |
| Primary care appointments | 60 (77) | 61 (73) |
| Community care visits | 10 (13) | 13 (15) |
| Outpatient appointments | 57 (73) | 62 (74) |
| Day case admissions | 1 (1) | 4 (5) |
| Inpatient (overnight) admissions | 1 (1) | 0 |
| Accident and emergency visits | 4 (5) | 8 (10) |
|  | Mean (SD) number of times services used | |
| GP | 2.2 (2.2) n=41 | 2.3 (2.0) n=35 |
| Physiotherapist (community) | 1 (0) n=4 | 1.3 (0.5) n=10 |
| Physiotherapist (outpatient) | 2.1 (1.9) n=8 | 3.0 (3.1) n=19 |
| Rheumatology (outpatient) | 1.8 (1.1) n=47 | 2.1 (1.4) n=53 |
| Occupational therapist (outpatient) | 1.6 (0.8) n=32 | 1.6 (0.8) n=27 |
| ^a^ Mean number of visits calculated for those reporting any visits only. | | |

**Supplementary Table S5: Results of incremental cost-effectiveness analysis for intervention versus control gloves: sensitivity analyses.**

|  | **Net cost**  **(95% CI)** | **Net QALY**  **(95% CI)** | **ICER (£/QALY)** | **Probability intervention is cost effective versus control if WTPT^a^ =** | | |
| --- | --- | --- | --- | --- | --- | --- |
| Primary analysis | | | | **£20,000/ QALY** | **£30,000/ QALY** | **£60,000/ QALY** |
| Complete cases (ITT) (n=151) | £251  (106, 396) | 0.003  (-0.017, 0.023) | £83,700/QALY | 0.19 | 0.29 | 0.44 |
| Sensitivity analysis - alternative costs (added/removed in turn) | | | | **£20,000/ QALY** | **£30,000/ QALY** | **£60,000/ QALY** |
| Including training cost of £10/participant | £262  (117, 408) | 0.003  (-0.017, 0.023) | £87,463/QALY | 0.17 | 0.28 | 0.43 |
| Including cost of glove review with OT^b^ | £261  (116, 407) | 0.003  (-0.017, 0.023) | £87,040/QALY | 0.17 | 0.29 | 0.44 |
| Alternative glove fitting cost^b^ | £194  (51, 337) | 0.003  (-0.017, 0.023) | £64,650/QALY | 0.26 | 0.37 | 0.48 |
| Sensitivity analysis – sample included in the analysis | | | | | | |
| Per protocol analysis (n=121)^c^ | £293  (18, 568) | 0.005  (-0.017, 0.026) | £58,526/QALY | 0.21 | 0.33 | 0.50 |
| Sensitivity analysis - alternative outcome measure | | | | **£1,000/ point** | **£10,000/ point** | **£20,000/ point** |
| Dominant hand pain (n=151) | £251  (106, 396) | 0.24  (-0.33, 0.82) | £1046 to improve by 1 point | 0.49 | 0.77 | 0.78 |
| ICER = incremental cost-effectiveness ratio; WTPT=willingness to pay threshold; ITT = intention to treat  Covariates costs: pre-baseline costs, stratification variable  Covariates QALYs: baseline health status, stratification variable  ^a^ willingness to pay thresholds based on 10,000 bootstrap simulations  Note: whole £ reported in table but ICERs calculated including pence  ^b^ based on cost of Band 5 OT time (£34/hour; PSSRU (2016), Unit Costs of Health and Social Care) and duration of individual appointments as reported by OTs  ^c^ excluding participants who were not treated as per the trial protocol  The training cost was calculated on the basis of two hours working time for the 32 OTs who attended training. This cost was divided by the number of participants in the intervention arm (n=103). This cost was then halved as it would only have been necessary to train half the number of OTs just to fit the intervention gloves. This gave a cost of £10 per person in the intervention group. | | | | | | |

| **-**  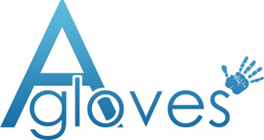 | 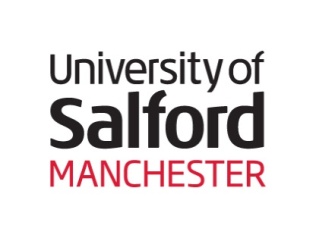 |
| --- | --- |

| **A-GLOVES: 12 week Follow-up Questionnaire**  **Office Use**:  **Site Number Participant ID:**   \|  \|  \|  \|  \|  \|  \| \| --- \| --- \| --- \| --- \| --- \| --- \|   **Centre Name: _______________________________________**  **PIN*:**   \|  \|  \|  \| \| --- \| --- \| --- \|     ***UoS use only:**  Participant contacted for missing data Yes  No  Participant contacted for minimal data only Yes  No |
| --- | --- | --- | --- | --- | --- | --- | --- | --- | --- |

| **Testing Arthritis Gloves in**  **Rheumatoid / Inflammatory Arthritis** |
| --- |

Thank you for taking part in this study. It will be a great help to us if you can complete and return this questionnaire within the **next week**.

It may take you about 30 minutes to fill in, depending on how much time you wish to spend on it. As you fill it out, don’t spend too much time on any one question. There are no right or wrong answers.

There are **four** sections:

1. Information about your hand pain and stiffness
2. The same four published questionnaires you completed at the start of this study to capture the impact of your condition on your daily activities and quality of life.
3. Information about any changes occurring in the last 12 weeks and your views about the arthritis gloves.
4. Your use of NHS services.

Please ensure you answer each question.

We also want to remind you that your answers will be kept completely confidential. Therefore, your answers will in no way affect your medical care.

**Instructions:**

For most of the questions all you need to do is:

a) **Circle the number** that best reflects your opinions. For example:

| ***Are you able to:*** | ***Never*** | ***Sometimes*** | ***Fairly often*** | ***Often*** | ***Always*** |
| --- | --- | --- | --- | --- | --- |
| Manage your everyday activities as you wish to | 1 | 2 |  | 4 | 5 |

**b) Or tick boxes.** For example,

Please describe any pain you have in your hands during your daily activities?

| **Not at all** | **Slightly** | **Moderately** | **Quite a bit** | **Extremely** |
| --- | --- | --- | --- | --- |
|  |  | **✓** |  |  |

If you need help filling in this questionnaire, please feel free to discuss this with a relative or friend. But please make sure the answers are your own views.

**We can be contacted on:**

Dr Yeliz Prior (Trial Manager): 0161 295 0211 or 07471 826 719 – y.prior@salford.ac.uk

Please check that you answer all questions and that no pages are missed by accident.

**Thank you for your assistance.**

**Please complete and return in the PRE-PAID envelope provided to:**

A-Gloves Trial Data Co-ordinator
Lancashire Clinical Trials Unit
Brook Building, Room BB418
University of Central Lancashire
Preston
PR1 2HE.

**Date of Completion: / /**

D D M M M Y Y Y Y

.g: 04 JUL 2016)

**Your Initials:**

**Your Date of Birth: / /**

D D M M M Y Y Y Y

**PART ONE:**

1. Are you: (please circle)

1. Right-handed
2. Left-handed
3. Both

2 Please rate your **RIGHT** hand condition at present: (Please **tick** one option)

| **Very Good** | **Good** | **Moderate** | **Severe** | **Very Severe** |
| --- | --- | --- | --- | --- |
|  |  |  |  |  |

**3** Please rate your **LEFT** hand condition at present: (Please **tick** one option)

| **Very Good** | **Good** | **Moderate** | **Severe** | **Very Severe** |
| --- | --- | --- | --- | --- |
|  |  |  |  |  |

**Please describe your hand pain:**

For the following questions we ask you to **CIRCLE** the number below the line which best reflects your situation **on a typical day in the last week**.

4. **In your RIGHT hand:** during the **daytime**, when doing moderate hand activities, e.g. housework, cooking, DIY, gardening:

0 1 2 3 4 5 6 7 8 9 10

No pain Severe pain

5. **In your RIGHT hand**, when you are resting during the day?

0 1 2 3 4 5 6 7 8 9 10

No pain Severe pain

6. **In your** **RIGHT hand**, during the night:

0 1 2 3 4 5 6 7 8 9 10

No pain Severe pain

7. **In your LEFT hand:** during the **daytime**, when doing moderate hand activities, e.g. housework, cooking, DIY, gardening:

0 1 2 3 4 5 6 7 8 9 10

No pain Severe pain

8. **In your LEFT hand**, when you are resting during the day?

0 1 2 3 4 5 6 7 8 9 10

No pain Severe pain

9. In your **LEFT hand**, during the night:

0 1 2 3 4 5 6 7 8 9 10

No pain Severe pain

**Please describe your hand stiffness:**

10. How long does any early morning stiffness in your hands last (hours/ minutes)? If you do not have early morning stiffness, put “0”:

_____(hour/s)_______ (minutes) (e.g. 1 hour 30 minutes)

11 Early morning stiffness **In your** **RIGHT** hand:

0 1 2 3 4 5 6 7 8 9 10

No stiffness Severe stiffness

12 Early morning stiffness **In your LEFT** hand:

0 1 2 3 4 5 6 7 8 9 10

No stiffness Severe stiffness

13. How are your hands in comparison to 3 months ago (i.e. before you received your arthritis gloves from the OT)? (Please tick one option below)

| **Much better** | **Better** | **No change** | **Worse** | **Much worse** |
| --- | --- | --- | --- | --- |
|  |  |  |  |  |

**PART TWO:**

1. **Measure of Activity Performance in the Hand**

This is an assessment of how you use your hands when doing everyday activities. Please tick the answer that best describes your ability to do the activities the last time you did them. If you use a gadget, please tick the answer that best describes your ability when using this.

|  | **No difficulty** | **Some difficulty** | **Great difficulty** | **Not able to do** |
| --- | --- | --- | --- | --- |

| 1. Buttoning buttons |  |  |  |  |
| --- | --- | --- | --- | --- |
| 1. Putting on socks or tights |  |  |  |  |
| 1. Tying shoelaces |  |  |  |  |
| 1. Squeezing out of tubes (e.g. toothpaste) |  |  |  |  |
| 1. Brushing teeth |  |  |  |  |
| 1. Wiping yourself after using the toilet |  |  |  |  |
| 1. Opening screw top bottles |  |  |  |  |
| 1. Opening cans (any type) |  |  |  |  |
| 1. Opening jam jars |  |  |  |  |
| 1. Slicing bread or cheese using a knife |  |  |  |  |
| 1. Peeling raw vegetables |  |  |  |  |
| 1. Stirring food in a pan |  |  |  |  |
| 1. Wringing out cloths |  |  |  |  |
| 1. Carrying shopping bags |  |  |  |  |
| 1. Writing by hand |  |  |  |  |
| 1. Typing on a computer |  |  |  |  |
| 1. Pushing with hands when getting up from a chair |  |  |  |  |
| 1. Carrying heavy objects like suitcases and bags (over 5kg/ 10 lbs) |  |  |  |  |

**B MICHIGAN HAND OUTCOMES QUESTIONNAIRE**

**Instructions**: This survey asks for your views about your hands and your health. This information will help keep track of how you feel and how well you are able to do your usual activities.

Answer ***EVERY*** question by marking the answer as indicated. If you are unsure about how to answer a question, please give the best answer you can.

I The following questions refer to the function of your hand(s)/wrist(s) ***during the past week***. (Please circle one answer for each question). Please answer ***EVERY*** question, even if you do not experience any problems with the hand and/or wrist.

- 1. The following questions refer to your ***right*** hand/wrist.

|  | **Very Good** | **Good** | **Fair** | **Poor** | **Very Poor** |
| --- | --- | --- | --- | --- | --- |
| 1. Overall, how well did your ***right*** hand work? | 1 | 2 | 3 | 4 | 5 |
| 2. How well did your ***right*** fingers move? | 1 | 2 | 3 | 4 | 5 |
| 3. How well did your ***right*** wrist move? | 1 | 2 | 3 | 4 | 5 |
| 4. How was the strength in your ***right*** hand? | 1 | 2 | 3 | 4 | 5 |
| 5. How was the sensation (feeling) in your ***right*** hand? | 1 | 2 | 3 | 4 | 5 |

Copyright 1998 the Regents of the University of Michigan.

All rights reserved.

| **B.** The following questions refer to your ***left*** hand/wrist. | | | | | |
| --- | --- | --- | --- | --- | --- |
|  | **Very Good** | **Good** | **Fair** | **Poor** | **Very Poor** |
| 1. Overall, how well did your ***left*** hand work? | 1 | 2 | 3 | 4 | 5 |
| 2. How well did your ***left*** fingers move? | 1 | 2 | 3 | 4 | 5 |
| 3. How well did your ***left*** wrist move? | 1 | 2 | 3 | 4 | 5 |
| 4. How was the strength in your ***left*** hand? | 1 | 2 | 3 | 4 | 5 |
| 5. How was the sensation (feeling) in your ***left*** hand? | 1 | 2 | 3 | 4 | 5 |

1. The following questions refer to the ability of your hand(s) to do certain tasks during the past week. (Please circle one answer for each question). If you do not do a certain task, please estimate the difficulty with which you would have in performing it.
   1. How difficult was it for you to perform the following activities using your ***right hand***?

|  | **Not at all difficult** | **A Little Difficult** | **Somewhat Difficult** | **Moderately Difficult** | **Very Difficult** |
| --- | --- | --- | --- | --- | --- |
| 1. Turn a door knob | 1 | 2 | 3 | 4 | 5 |
| 1. Pick up a coin | 1 | 2 | 3 | 4 | 5 |
| 1. Hold a glass of water | 1 | 2 | 3 | 4 | 5 |
| 1. Turn a key in a lock | 1 | 2 | 3 | 4 | 5 |
| 1. Hold a frying pan | 1 | 2 | 3 | 4 | 5 |
| - 1. How difficult was it for you to perform the following activities using your ***left hand***? | | | | | |
|  | **Not at all difficult** | **A Little Difficult** | **Somewhat Difficult** | **Moderately Difficult** | **Very Difficult** |
| 1. Turn a door knob | 1 | 2 | 3 | 4 | 5 |
| 1. Pick up a coin | 1 | 2 | 3 | 4 | 5 |
| 1. Hold a glass of water | 1 | 2 | 3 | 4 | 5 |
| 1. Turn a key in a lock | 1 | 2 | 3 | 4 | 5 |
| 1. Hold a frying pan | 1 | 2 | 3 | 4 | 5 |

| - 1. How difficult was it for you to perform the following activities using ***both of your hands****?* | | | | | |
| --- | --- | --- | --- | --- | --- |
|  | **Not at all difficult** | **A Little Difficult** | **Somewhat Difficult** | **Moderately Difficult** | **Very Difficult** |
| 1. Open a jar | 1 | 2 | 3 | 4 | 5 |
| 1. Button a shirt/ blouse | 1 | 2 | 3 | 4 | 5 |
| 1. Eat with a knife/ fork | 1 | 2 | 3 | 4 | 5 |
| 1. Carry a grocery bag | 1 | 2 | 3 | 4 | 5 |
| 1. Wash dishes | 1 | 2 | 3 | 4 | 5 |
| 1. Wash your hair | 1 | 2 | 3 | 4 | 5 |
| 1. Tie shoelaces/ knots | 1 | 2 | 3 | 4 | 5 |

| 1. The following questions refer to how you did in your ***normal work*** (**including** both housework and school work) during the ***past four weeks*** (Please circle one answer for each question) | | | | | |
| --- | --- | --- | --- | --- | --- |
|  | **Always** | **Often** | **Sometimes** | **Rarely** | **Never** |
| 1. How often were you unable to do your work because of problems with your hand(s)/ wrist(s) | 1 | 2 | 3 | 4 | 5 |
| 1. How often did you have to shorten your work day because of problems with your hand(s)/ wrist(s) | 1 | 2 | 3 | 4 | 5 |
| 1. How often did you have to take it easy at your work because of problems with your hand(s)/ wrist(s)? | 1 | 2 | 3 | 4 | 5 |
| 1. How often did you accomplish less in your work because of problems with your hand(s)/ wrist(s)? | 1 | 2 | 3 | 4 | 5 |
| 1. How often did you take longer to do the tasks in your work because of problems with your hand(s)/ wrist(s) | 1 | 2 | 3 | 4 | 5 |

1. The following questions refer to how ***pain*** you had in your hand(s)/ wrist(s) ***during the past week.***  (Please circle one answer for each question).
   1. The following questions refer to **pain** in your ***right*** hand/wrist.

1 How often did you have pain in your ***right*** hand/ wrist?

- - - 1. Always
      2. Often
      3. Sometimes
      4. Rarely
      5. Never

If you answered **never** to question **IV-A1** above, please **skip** the following questions and go to the next page.

2 Please describe the pain you had in your ***right*** hand/ wrist

1. Very mild
2. Mild
3. Moderate
4. Severe
5. Very severe

|  | **Always** | **Often** | **Sometimes** | **Rarely** | **Never** |
| --- | --- | --- | --- | --- | --- |
| 1. How often did the pain in your ***right*** hand/ wrist interfere with your sleep? | 1 | 2 | 3 | 4 | 5 |
| 1. How often did the pain in your ***right*** hand/ wrist interfere with your daily activities (such as eating or bathing)? | 1 | 2 | 3 | 4 | 5 |
| 1. How often did the pain in your ***right*** hand/ wrist make you unhappy? | 1 | 2 | 3 | 4 | 5 |

- 1. The following questions refer to **pain** in your ***left*** hand/wrist.

1 How often did you have pain in your ***left*** hand/ wrist?

- - - 1. Always
      2. Often
      3. Sometimes
      4. Rarely
      5. Never

If you answered **never** to question **IV-B1** above, please **skip** the following questions and go to the next page.

2 Please describe the pain you had in your ***left*** hand/ wrist

1. Very mild
2. Mild
3. Moderate
4. Severe
5. Very severe

|  | **Always** | **Often** | **Sometimes** | **Rarely** | **Never** |
| --- | --- | --- | --- | --- | --- |
| 1. How often did the pain in your ***left*** hand/ wrist interfere with your sleep? | 1 | 2 | 3 | 4 | 5 |
| 1. How often did the pain in your ***left*** hand/ wrist interfere with your daily activities (such as eating or bathing)? | 1 | 2 | 3 | 4 | 5 |
| 1. How often did the pain in your ***left*** hand/ wrist make you unhappy? | 1 | 2 | 3 | 4 | 5 |

1. **A.** The following questions refer to the appearance (look) of your ***right*** hand during the past week. (Please circle one answer for each question).

|  | **Strongly Agree** | **Agree** | **Neither Agree nor Disagree** | **Disagree** | **Strongly Disagree** |
| --- | --- | --- | --- | --- | --- |
| 1. I am satisfied with the appearance (look) of my ***right*** hand. | 1 | 2 | 3 | 4 | 5 |
| 1. The appearance (look) of my ***right*** hand sometimes made me uncomfortable in public. | 1 | 2 | 3 | 4 | 5 |
| 1. The appearance (look) of my ***right*** hand made me depressed. | 1 | 2 | 3 | 4 | 5 |
| 1. The appearance (look) of my ***right*** hand interfered with my normal social activities. | 1 | 2 | 3 | 4 | 5 |

| 1. The following questions refer to the appearance (look) of your ***left*** hand during the past week (Please circle one answer for each question). | | | | | |
| --- | --- | --- | --- | --- | --- |
|  | **Strongly Agree** | **Agree** | **Neither Agree nor Disagree** | **Disagree** | **Strongly Disagree** |
| 1. I am satisfied with the appearance (look) of my ***left*** hand. | 1 | 2 | 3 | 4 | 5 |
| 1. The appearance (look) of my ***left*** hand sometimes made me uncomfortable in public. | 1 | 2 | 3 | 4 | 5 |
| 1. The appearance (look) of my ***left*** hand made me depressed. | 1 | 2 | 3 | 4 | 5 |
| 1. The appearance (look) of my ***left*** hand interfered with my normal social activities. | 1 | 2 | 3 | 4 | 5 |

1. **A.** The following questions refer to your satisfaction with your ***right*** hand/wrist during the past week (Please circle one answer for each question).

|  | **Very Satisfied** | **Somewhat Satisfied** | **Neither satisfied no Dissatisfied** | **Somewhat Dissatisfied** | **Very Dissatisfied** |
| --- | --- | --- | --- | --- | --- |
| 1. Overall function of your ***right*** hand. | 1 | 2 | 3 | 4 | 5 |
| 1. Motion of the fingers in your ***right*** hand. | 1 | 2 | 3 | 4 | 5 |
| 1. Motion of your ***right*** wrist. | 1 | 2 | 3 | 4 | 5 |
| 1. Strength of your ***right*** hand. | 1 | 2 | 3 | 4 | 5 |
| 1. Pain level of your ***right*** hand. | 1 | 2 | 3 | 4 | 5 |
| 1. Sensation (feeling) of your ***right*** hand. | 1 | 2 | 3 | 4 | 5 |
| 1. The following questions refer to your satisfaction with your ***left*** hand/ wrist during the past week (Please circle one answer for each question). | | | | | |
|  | **Very Satisfied** | **Somewhat Satisfied** | **Neither satisfied no Dissatisfied** | **Somewhat Dissatisfied** | **Very Dissatisfied** |
| 1. Overall function of your ***left*** hand. | 1 | 2 | 3 | 4 | 5 |
| 1. Motion of the fingers in your ***left*** hand. | 1 | 2 | 3 | 4 | 5 |
| 1. Motion of your ***left*** wrist. | 1 | 2 | 3 | 4 | 5 |
| 1. Strength of your ***left*** hand. | 1 | 2 | 3 | 4 | 5 |
| 1. Pain level of your ***left*** hand. | 1 | 2 | 3 | 4 | 5 |
| 1. Sensation (feeling) of your ***left*** hand. | 1 | 2 | 3 | 4 | 5 |

**C Health Assessment Questionnaire**

We are interested in learning how your illness affects your ability to function in daily life. Please **circle** the one response that best describes your usual abilities **OVER THE PAST WEEK*.***

|  | ***Without ANY difficulty*** | ***With SOME difficulty*** | ***With MUCH difficulty*** | ***UNABLE to do*** |
| --- | --- | --- | --- | --- |
| **DRESSING AND GROOMING:**  **Are you able to:** |  |  |  |  |
| Dress yourself, including tying shoes and doing buttons? | 0 | 1 | 2 | 3 |
| Shampoo your hair? | 0 | 1 | 2 | 3 |
| **RISING: Are you able to:** |  |  |  |  |
| Stand up from an armless straight chair? | 0 | 1 | 2 | 3 |
| Get in and out of bed? | 0 | 1 | 2 | 3 |
| **EATING. Are you able to:** |  |  |  |  |
| Cut your meat/ food? | 0 | 1 | 2 | 3 |
| Lift a full cup or glass to your mouth? | 0 | 1 | 2 | 3 |
| Open a new milk carton (or soap powder)? | 0 | 1 | 2 | 3 |
| WALKING: Are you able to? |  |  |  |  |
| Walk outdoors on flat ground? | 0 | 1 | 2 | 3 |
| Climb up five steps? | 0 | 1 | 2 | 3 |

Please **tick any aids or devices** that you usually use for any of these activities:

_____Walking stick/cane _____Gadgets for dressing (eg button hook, long

_____Walking frame handled shoehorn, zipper pull etc)

_____Crutches _____ Built-up or special utensils

_____Wheelchair _____Special or built-up chair

Other (*please specify*)___________________________________________________________

Please **tick any categories for which you usually need help** from another person:

_____Dressing and grooming _____ Eating

_____Rising _____ Walking

Please **circle** the one response which best describes your usual abilities **OVER THE PAST WEEK:**

|  | ***Without ANY difficulty*** | ***With SOME difficulty*** | ***With MUCH difficulty*** | UNABLE to do |
| --- | --- | --- | --- | --- |
| **HYGIENE: Are you able to:** |  |  |  |  |
| Wash and dry your entire body? | 0 | 1 | 2 | 3 |
| Take a bath? | 0 | 1 | 2 | 3 |
| Get on and off the toilet? | 0 | 1 | 2 | 3 |
| **REACH. Are you able to:** |  |  |  |  |
| Reach and get down a 5lb (2kg) object (e.g. bag of potatoes) from just above your head? | 0 | 1 | 2 | 3 |
| Bend down to pick up clothing from the floor? | 0 | 1 | 2 | 3 |
| **GRIP. Are you able to:** |  |  |  |  |
| Open car doors? | 0 | 1 | 2 | 3 |
| Open jars which have been previously opened? | 0 | 1 | 2 | 3 |
| Turn taps on and off? | 0 | 1 | 2 | 3 |
| **ACTIVITIES. Are you able to:** |  |  |  |  |
| Run errands and shop? | 0 | 1 | 2 | 3 |
| Get in and out of a car? | 0 | 1 | 2 | 3 |
| Do chores such as vacuuming, housework or light gardening? | 0 | 1 | 2 | 3 |

Please **tick** any **aids or devices** that you usually use for any of these activities:

_____Raised toilet seat _____ Bath rail

_____Bath seat _____ Long handled appliances for reach

_____Jar opener (for jars previously opened)

Other (please specify)

____­­­­­­­­­­­­­­­­­­­­­­­___________________________________________________

Please **tick** any **categories for which you usually need help** from another person:

_____Hygiene _____Gripping and opening things

_____Reach _____Errands and housework

1. **Your health (EQ-5D-3L)**

Under **each** heading, please tick the **ONE** box that best describes your health **TODAY.**

| **MOBILITY** |  |
| --- | --- |
| I have no problems in walking about | □ |
| I have some problems in walking about | □ |
| I am unable to walk about | □ |
| **SELF CARE** |  |
| I have no problems washing or dressing myself | □ |
| I have some problems washing or dressing myself | □ |
| I am unable to wash or dress myself | □ |
| **USUAL ACTIVITIES** (e.g. work, study, housework, family or leisure activities) |  |
| I have no problems doing my usual activities | □ |
| I have some problems doing my usual activities | □ |
| I am unable to do my usual activities | □ |
| **PAIN/DISCOMFORT** |  |
| I have no pain or discomfort | □ |
| I have moderate pain or discomfort | □ |
| I have extreme pain or discomfort | □ |
| **ANXIETY/DEPRESSION** |  |
| I am not anxious or depressed | □ |
| I am moderately anxious or depressed | □ |
| I am extremely anxious or depressed | □ |

| To help people say how good or bad a health state is, we have drawn a scale (rather like a thermometer) on which the best state you can imagine is marked 100 and the worst state you can imagine is marked 0. |
| --- |
|  |
| We would like you to indicate on this scale how good or bad your own health is today, in your opinion. Please do this by drawing a line:  **from** the box below **to** whichever point on the scale indicates how good or bad your health state is today. |

Best imaginable health state

9 0

8 0

7 0

6 0

5 0

4 0

3 0

2 0

1 0

100

0

Your own health state today

Worst imaginable health state

**PART THREE:**

1. **In the last 3 months** (since completing the previous study questionnaire), have you…

Please TICK…

1. Had any medication changes or started any new drugs **for your arthritis**?

Yes  No

If yes: what changes were made?:

……………………………………………………………………………………………….

…………………………………………………………………………………………….

……………………………………………………………………………………

1. had a steroid injection?

Yes  No

1. Received any occupational therapy (APART from your glove provision and review appointments) or any physiotherapy treatment?

Yes  No

**If yes**, please state what this was and how long it lasted (*e.g. saw a physiotherapist 3 times for leg exercises; saw the OT once for help with everyday activities*)

2. In the last 3 months, have you worn any splints on your hands (apart from arthritis gloves)? (Please tick)

Yes  No

**If yes**: please tick which: (Please tick all that apply).

**Right hand Left hand**

| Resting splint |  |  |
| --- | --- | --- |
| Wrist splint |  |  |
| Finger splint |  |  |
| Thumb splint |  |  |
| Wrist and thumb support splint |  |  |

1. **Glove use during the day**
2. I was recommended to wear the glove(s) during the day (please tick): Yes  No
3. **In the last 4 weeks, during the day** on average, how many days a week have you worn your arthritis gloves?

*If you did not receive a glove for either your right or for your left hand, please circle “Not Given”*

| **RIGHT GLOVE** | *Not given* | 0 | 1 | 2 | 3 | 4 | 5 | 6 | 7 |
| --- | --- | --- | --- | --- | --- | --- | --- | --- | --- |
| **LEFT GLOVE** | *Not given* | 0 | 1 | 2 | 3 | 4 | 5 | 6 | 7 |

iii) On average, how many hours/ minutes did you wear your glove/s each day? *(If this varied, please give your best estimate overall for a typical day)*

Right glove _____hours _____minutes Left glove _____hours _____minutes

4 **Glove use during the night**

1. I was recommended to wear the glove(s) during the night (please tick): Yes  No
2. **In the last 4 weeks, during the night**, on average, how many nights a week have you worn your arthritis gloves?

*If you did not receive a glove for either your right or for your left hand, please circle “Not Given”*

| **RIGHT GLOVE** | *Not given* | 0 | 1 | 2 | 3 | 4 | 5 | 6 | 7 |
| --- | --- | --- | --- | --- | --- | --- | --- | --- | --- |
| **LEFT GLOVE** | *Not given* | 0 | 1 | 2 | 3 | 4 | 5 | 6 | 7 |

iii) On average, how many hours/minutes did you wear your glove/s each night? *(If this varied, please give your best estimate overall for a typical night).*

Right glove _______hours _____minutes Left glove _______hours ___minutes

5. Have you worn the arthritis gloves as advised by the therapist when you were given them?

Day-time: Yes  No

Night-time: Yes  No

6. Have you bought, or obtained, any other type of “arthritis” gloves from elsewhere? (*please tick*)

Yes  No

**If no, proceed to Question 7 over the page**

i) **In the last 4 weeks, during the day** on average, how many days a week have you worn the arthritis gloves you got for yourself?

If yes, what type these were? (*e.g. fingerless thermal gloves. Pease state the make of glove, if you know it*)

| **RIGHT GLOVE** | 0 | 1 | 2 | 3 | 4 | 5 | 6 | 7 |
| --- | --- | --- | --- | --- | --- | --- | --- | --- |
| **LEFT GLOVE** | 0 | 1 | 2 | 3 | 4 | 5 | 6 | 7 |

ii) **In the last 4 weeks, during the night**, on average, how many nights a week have you worn the arthritis gloves you got for yourself?

| **RIGHT GLOVE** | 0 | 1 | 2 | 3 | 4 | 5 | 6 | 7 |
| --- | --- | --- | --- | --- | --- | --- | --- | --- |
| **LEFT GLOVE** | 0 | 1 | 2 | 3 | 4 | 5 | 6 | 7 |

7. What is your opinion of the arthritis gloves **provided to you by the OT**? (Please tick)

Yes, they were of benefit

No, they were not of benefit

8. **If yes, you found the arthritis gloves of benefit,** please tick which of the following apply. You can tick more than one:

| Hands feel less painful in the day |  |  |
| --- | --- | --- |
| Hands feel less painful in the night |  |  |
| Hands feel less stiff |  |  |
| Gloves give comfort |  |  |
| Able to do things better- personal care/grooming |  |  |
| Able to do things better- household activities |  |  |
| Able to do things better- leisure/ social activities |  |  |
| Able to do things better- at work |  |  |
| Stronger hands/wrists |  |  |
| Gloves give support |  |  |
| Hands feel less swollen |  |  |
| Gloves give warmth |  |  |
| Hand(s) feel more flexible |  |  |
| Using a keyboard (e.g. computer/laptop) |  |  |
| Using a tablet computer (e.g.i-Pad) |  |  |
| Using a mobile/ smart phone |  |  |
| Sleep better |  |  |
| Take fewer painkillers |  |  |
| More confident doing activities |  |  |
| Less frustrated doing activities |  |  |
| Improved mood |  |  |
| Feel better overall |  |  |
|  |  |  |
| **If you found any other benefits or have any other comments, please state:** | | |

**9. If you had any problems wearing the gloves,** please tell us why?

| Day-time: |
| --- |
| Night-time: |

1. If you **stopped** wearing the arthritis gloves completely (in the day and/or at night) which were provided by the OT, please state why:

| I stopped wearing the gloves completely during the day because: |
| --- |
| I stopped wearing the gloves completely during the night because: |

10. Would you want to continue to wear the arthritis gloves provided by the OT?

Yes  No

11. In future, would you be willing to buy replacement arthritis gloves of the same type you received from the OT?

Yes  No

**PART FOUR:**

**Please tell us about your use of NHS and Social Services:**

**1. Your use of hospital in-patient services**

1. Have you had any **planned hospital** **overnight stays** in the last **3 months?** *(please tick)*

Yes  No  If no, continue to question 2.

1. If **YES**, please list below any **planned** admissions you had to hospital involving overnight stays and the number of days you were in hospital each time. Examples of departments you might have been admitted to are, for example, a “Medical ward” or “Orthopaedic ward”; or a reason might be e.g. “Gall stone operation” or “hip joint replacement.”

*(Please do* ***not*** *include any hospital out-patient appointments, day hospital appointments, accident and emergency services or admissions as a result of attending A&E here. We will ask you for information about these in questions 2, 3, and 4).*

| **Department - please give type of department or reason you were admitted** | **Name of Hospital** | **Admission date**  **(month/year)** | **Length of stay in hospital (i.e. number of nights)** |
| --- | --- | --- | --- |
|  |  | __ / ____ |  |
|  |  | __ / ____ |  |
|  |  | __ / ____ |  |
|  |  | __ / ____ |  |
|  |  | __ / ____ |  |
|  |  | __ / ____ |  |
|  |  | __ / ____ |  |

**2. Your use of hospital out-patient appointments**

1. Have you attended any **planned hospital out-patient appointments** lasting 4 hours or less in the last **3 months?** *(please tick)*

Yes  No  If no, continue to question 3.

1. If **YES**, please tell us about the department or specialty and the number of appointments which **lasted 4 hours or less***.* If you did not have appointments at some, please put “0” against those services.

*(Please do not include any hospital in-patient admissions, day hospital appointments, accident and emergency services or admissions as a result of attending A&E here. We ask you for information about these in questions 1, 3 and 4).*

| **Department or specialty** | **Number of visits to each department in the last 3 months** |
| --- | --- |
| *Please list each type of department/clinic or specialty separately and tell us the number of visits for this hospital department/clinic or specialty (e.g. Rheumatology, Orthopaedics, Diabetology, hospital-based occupational therapy, hospital-based physiotherapy, hospital blood tests).*  *If you did not attend that department, put “0” under number of visits* | |
| Rheumatology |  |
| Occupational Therapy |  |
| Physiotherapy |  |
| Other (please state): |  |
|  |  |
|  |  |

**3. Your use of day hospital appointments**

1. Have you attended any day or hospital outpatient appointments which lasted for **more than 4 hours (but not overnight)** during the last **3 months**?(please tick)

Yes  No  If no, please go to question 4

1. If **YES**, tell us about the department or specialty and the number of appointments lasting **more than 4 hours (but not overnight).**

*(Please do not include any hospital in-patient admissions, out-patient hospital appointments under 4 hours, accident and emergency services or admissions resulting from going to A&E here. We ask you for information about these in questions 1, 2 and 4).*

| **Department or specialty** | **Number of visits to each department in the last 3 months** |
| --- | --- |
| *Please list each type of department/clinic or specialty separately and tell us the number of visits to this department/clinic or specialty (e.g. minor surgery, dialysis, chemotherapy, other diagnostic procedures)* | |
|  |  |
|  |  |
|  |  |
|  |  |
|  |  |

**4. Your use of accident and emergency (A&E) services**

1. Have you attended an Accident and Emergency (A&E) unit during the last **3 months**?

Yes  No  If no, please go to question 5.

1. If **YES**, first tell us about the number of A & E visits you had which did **not** lead to a hospital admission.

*(Please do not include any planned hospital in-patient admissions, hospital out-patient appointments or day hospital appointments here. We ask you for information about these in questions 1,2 and 3).*

| For Accident and Emergency visits **not** leading to in-patient admission: |
| --- |
| **Number of visits to A&E during the last 3 months?** |
|  |

1. Were you admitted into a hospital as an **in-patient** directly from the Accident and Emergency (A&E) unit during the last **3 months**?

Yes  No  If no, please go to question 5

1. **If yes**, please tell us which department/ for what reason you were admitted, where and when you were admitted each time. Examples of departments you might have been admitted to are, for example, a “Medical ward” or “Surgical ward”; or a reason might be e.g. “heart failure” or “fall.”

| **Department - please give type of department or reason you were admitted** | **Name of Hospital** | **Admission date**  **(month/year)** | **Length of stay in hospital (i.e. number of nights)** |
| --- | --- | --- | --- |
|  |  | __ / ____ |  |
|  |  | __ / ____ |  |
|  |  | __ / ____ |  |
|  |  | __ / ____ |  |
|  |  | __ / ____ |  |

**5. Your use of primary and community based health services**

1. Have you used any of the services below in the last **3 months?**

Yes  No  If no, please go to the next page.

1. If **YES**, please state the number of appointments for each. If you did not have appointments at some, please put “0” against those services.

| **GP practice services** | **Number of visits in the last 3 months?** |
| --- | --- |
| GP (at the surgery/practice) |  |
| GP (at your home) |  |
| Practice Nurse (at the surgery) |  |
| Nurse (at your home) |  |
| Counsellor *or* mental health worker |  |
|  |  |

| **Other physical care services** (e.g. minor illness or injury, diagnostic test, blood sample test) | **Number of visits in the last 3 months?** |
| --- | --- |
| Walk-in-centre |  |
| Blood test at the surgery/ practice |  |
| Other *(please specify)* |  |
|  |  |
|  |  |

1. **Your use of primary and community based health services (continued)**
2. Have you used any of the following services in the last **3 months?**

Yes  No  .

1. If YES, please state the number of appointments with/ visits from each. If you did not have appointments with/ visits from some, please put “0” against those services.

| **Other community / social support services**  *(e.g. social worker, home help, care worker, occupational or physiotherapist)*  *(please specify)* | **Total visits in the last 3 months?** |
| --- | --- |
| Occupational therapist (community-based or from the Social Services) |  |
| Physiotherapist (community-based) |  |
| Care worker |  |
| Home help |  |
| Social worker |  |
| Other *(please specify):* |  |
|  |  |
|  |  |
|  |  |

*Thank you for completing these questions!*

Many thanks indeed for taking the time to answer this, which we very much appreciate. Please could you go back and check you have not left out a page or any questions by mistake.

**Please complete and return in the PRE-PAID envelope provided to:**

A-Gloves Trial Data Co-ordinator
Lancashire Clinical Trials Unit
Brook Building, Room BB418
University of Central Lancashire
Preston
PR1 2HE.
